# Manifestations of Renal Impairment in Fructose-induced Metabolic Syndrome

**DOI:** 10.7759/cureus.1826

**Published:** 2017-11-07

**Authors:** Kameliya Bratoeva, George S Stoyanov, Albena Merdzhanova, Mariya Radanova

**Affiliations:** 1 Department of Physiology and Pathophysiology, Division of Pathophysiology, Medical University – Varna; 2 Department of Anatomy and Cell Biology, Medical University – Varna; 3 Department of Chemistry, Medical University – Varna; 4 Department of Biochemistry, Molecular Medicine and Nutrigenomics, Medical University – Varna

**Keywords:** amyloid a, metabolic syndrome, chronic kidney disease, fructose

## Abstract

Introduction

International studies show an increased incidence of chronic kidney disease (CKD) in patients with metabolic syndrome (MS). It is assumed that the major components of MS - obesity, insulin resistance, dyslipidemia, and hypertension - are linked to renal damage through the systemic release of several pro-inflammatory mediators, such as uric acid (UA), C-reactive protein (CRP), and generalized oxidative stress. The aim of the present study was to investigate the extent of kidney impairment and manifestations of dysfunction in rats with fructose-induced MS.

Methods

We used a model of high-fructose diet in male Wistar rats with 35% glucose-fructose corn syrup in drinking water over a duration of 16 weeks. The experimental animals were divided into two groups: control and high-fructose drinking (HFD). Serum samples were obtained from both groups for laboratory study, and the kidneys were extracted for observation via light microscopy examination.

Results

All HFD rats developed obesity, hyperglycemia, hypertriglyceridemia, increased levels of CRP and UA (when compared to the control group), and oxidative stress with high levels of malondialdehyde and low levels of reduced glutathione. The kidneys of the HFD group revealed a significant increase in kidney weight in the absence of evidence of renal dysfunction and electrolyte disturbances. Under light microscopy, the kidneys of the HFD group revealed amyloid deposits in Kimmelstiel-Wilson-like nodules and the walls of the large caliber blood vessels, early-stage atherosclerosis with visible ruptures and scarring, hydropic change (vacuolar degeneration) in the epithelial cells covering the proximal tubules, and increased eosinophilia in the distant tubules when compared to the control group.

Conclusion

Under the conditions of a fructose-induced metabolic syndrome, high serum UA and CRP correlate to the development of early renal disorders without a clinical manifestation of renal dysfunction. These phenomena are of particular importance for assessing the risk of developing future CKD.

## Introduction

Metabolic syndrome (MS) has reached epidemic proportions on a global scale with serious consequences for human health with high mortality, including cardiovascular damage, non-alcoholic fatty liver disease, and increased incidence of chronic kidney disease (CKD) [1-2]. The increasing incidence is associated with alteration in eating habits, low physical activity, increased consumption of high-calorie foods, and drinks supplemented with fructose [3]. At present, the International Diabetes Federation has proposed that MS criteria include at least three of the four major components of the disease - abdominal obesity, insulin resistance, dyslipidemia, and arterial hypertension. CKD is defined as a sustained decrease in glomerular filtration rate and the presence of structural or functional abnormalities of the kidneys, determined by urine analysis, biopsy, or diagnostic imaging [4]. Accumulating data show that MS and CKD share many important cardiometabolic risk factors and some common pathogenetic mechanisms. Disease-causing obesity, hyperglycaemia, atherogenic dyslipidemia, endothelial dysfunction, and stimulation of vasoconstrictor systems in MS are believed to be associated with renal impairment by systemic release of glycated products, free radicals, and some pro-inflammatory mediators, such as uric acid (UA) and C-reactive protein (CRP) [5-7]. In diabetic patients with diabetic nephropathy and in experimental models of diabetes, there have been reports of glomerular depositions of serum amyloid A (a potent proinflammatory protein), which stimulate glomerular and tubulointerstitial inflammation, and interstitial fibrosis [8]; also, in people with atherosclerosis, the amyloid is expressed in the vascular wall, causing inflammation and endothelial dysfunction [6].

Nevertheless, strategies for early identification and treatment of people with MS who develop CKD are still insufficiently effective due to the fact that few studies have been conducted in this field. The screening of CKD includes microalbuminuria and a glomerular filtration rate test among patients with arterial hypertension, diabetes mellitus, and specific criteria for different age groups; however, its role in detecting early renal manifestations in MS patients remains controversial [4]. In parallel, CKD is a common disease that is a risk factor for cardiovascular disease, kidney failure, and death. This emphasizes the need for further studies, assessing the presence and extent of renal impairment in MS patients at risk, and identifying informative biomarkers to predict the risk of future development and progression of CKD.

The goal of this study was to investigate morphological changes in the kidneys and manifestations of dysfunction in rats with fructose-induced MS.

## Materials and methods

Animal models

The experimental procedures were approved by the Home Office for Care and Use of Laboratory Animals and performed with a strong consideration for ethics of animal experimentation according to the International Guiding Principles for Animal Research approved in Bulgaria.

Male Wistar rats were housed in a 20 ± 2ºC room temperature setting with a standard 12-hour light and dark cycle. The rats received a standard diet and water at will. The standard diet was composed of starch - 50%, protein - 20%, fat - 4.5%, cellulose - 5%, and a standard vitamin and mineral mix. At the beginning of the experiment, the body weight of all rats was 140 - 180 grams. After an acclimation of two weeks, the animals were randomly assigned to two groups of seven each: a high-fructose drinking (HFD) group on a diet including 35% glucose-fructose corn syrup in drinking water and a control group (C) only on the standard diet. The period of the experiment was 16 weeks.

After the 16 weeks had passed, the body mass of each animal from both groups was measured and blood samples were collected from the tail vein, after pre-narcosis with thiopental, 15 mg/kg intraperitoneally. Serum samples were obtained from the drawn blood and used to determine some of the biochemical indicators on the same day, whilst other markers were evaluated in another time period from the remaining frozen serum.

Under continued general anesthesia with thiopental, 30 mg/kg intravenously, a laparotomy was conducted, and the kidneys were extracted and weighed. All procedures were performed at 4 - 8ºC.

Histological study

The kidneys were formed in sections with dimensions 10 mm long and 10 mm wide and fixed in 10% formaldehyde (pH = 7.0). After the fixation in formalin, the tissue sections were embedded in paraffin. The kidneys were then examined histopathologically via light microscopy using four different stains – hematoxylin and eosin (H&E), an experimental amyloid stain, Lugol’s iodine for staining amyloid (LISA), a standard amyloid stain, Congo red, and Van-Gieson for fibrin deposits. The H&E, Congo red, and Van-Gieson stains were carried out under standardized conditions and protocols.

For the LISA stain, the formalin-fixed paraffin embedded tissue sections were cut in 10 µm thick slides and fixed on standard glass slides. The sections were then stained for five minutes with a ready-to-use solution of Lugol’s iodine and then dipped in the standard concentration of acetic acid for 30 seconds.

Biochemical and clinical laboratory analyses

The serum concentrations of triglycerides (TGs), glucose, creatinine, total protein, urea, uric acid, and electrolyte tests were determined using a commercially available kit on the automatic analyzer Olympus AU640 (Olympus Corp., Center Valley, PA). Serum CRP levels were determined by the immunoturbidimetric method using Olympus-400 analyzer (Olympus Corp., Center Valley, PA) in g/L.

Reduced glutathione (GSH) levels in sera were defined as the total number of sulfhydryl groups (SH groups) in the sample. SH groups were determined by the method of Hu (1994), based on the absorption of the color complex between thiol groups and 5,5'-dithiobis-(2- nitrobenzoic acid (DTNB) (Merck, Darmstadt, Germany) at 412 nm [9]. Standard solutions of reduced glutathione were used to calculate the concentration of thiol groups.

Membrane lipid peroxidation was assayed using malondialdehyde (MDA) measured by its thiobarbituric acid (TBA) (Merck, Darmstadt, Germany) reactivity in sera using the method detailed by Porter, et al. [10]. Results were determined using the extinction coefficient of MDA–TBA complex at 532 nm = 1.56 – 10-5 cm-1 M-1 solution and calculated in (mmol/L).

Statistical analyses

All results were expressed as means ± standard error of the mean (SEM). The statistical significance of the studied parameters was analyzed using Student’s t-test. P values of less than 0.05 were regarded as significant. All tests were two-tailed. The statistical procedure was performed with GraphPad In Stat software (GraphPad Software, Inc., La Jolla, CA).

## Results

The body weight of the experimental animals was measured weekly with consistent increases in all groups through the end of the experiment. The final results showed a significant progression towards obesity in the HFD group as compared to the control group with more than a 50% increase in body weight (p < 0.05) and a 26% increase in kidney weight (p < 0.05) (Table 1).

**Table 1:** Markers of Metabolic Abnormalities, Oxidative Stress, and Inflammation Measured in the Control and Experimental Groups C: control group rats; HFD: high-fructose drinking rats; TG: triglycerides; MDA: malondialdehyde; GSH: reduced glutathione; UA: uric acid; CRP: c-reactive protein

Groups	C	HFD	P-value
Body weight (g)			
Initial	143.9 ± 10.6	162.0 ± 7.1	p > 0.05
Final	250.0 ± 8.1	366.0 ± 21.5^*^	p < 0.05
Kidney weight (g)	1.8 ± 0.08	2.2 ± 0.16^*^	p < 0.05
Serum glucose (mmol/L)	8.14 ± 0.31	10.72 ± 0.56^**^	p < 0.005
Serum TGs (mmol/L)	1.076 ± 0.07	1.704 ± 0.22^*^	p < 0.05
Serum MDA (mmol/L)	2.12 ± 0.02	2.28 ± 0.04^**^	p < 0.005
Serum GSH (mmol/L)	157.5 ± 10.04	241.9 ± 10.8^***^	p < 0.0005
Serum UA (mmol/L)	80.94 ± 5.5	315.9 ± 48.2**	p < 0.005
Serum CRP (g/L)	7.155 ± 0.12	14.87 ± 0.31^**^	p < 0.005

In the HFD group, the levels of glucose (p < 0.005), CRP (p < 0.005), and TGs (p < 0.05) were significantly higher than those in the control group (Table 1).

The results showed the presence of lipid peroxidation, an imbalance in antioxidative protection detected with significantly higher levels of MDA (p < 0.005), and a significant reduction (over 50%) of SH groups (p < 0.0005) in the HFD versus the control group (Table 1).

The results indicated normal renal function without electrolyte disturbances and variation in serum creatinine, total protein (TP), and urea between the two groups, while uric acid (UA) levels were significantly increased in the HFD group when compared to the control group (p < 0.005) (Tables 1-2).

**Table 2:** Indicators for Impairment of Renal Function and Electrolyte Disturbances in the Control and Experimental Groups Mean levels ± standard error of the mean (SEM), n=7 C: control group rats; HFD: high-fructose drinking rats; TP: total protein; Na: sodium; K: potassium; Cl: chloride; Ca: calcium; P: phosphorus

	TP g/l	Na mmol/l	K mmol/l	Cl mmol/l	Creatinine µmol/l	Urea mmol/l	Ca mmol/l	P mmol/l
HFD	74.5±0.5	142.1±0.05	4.01±0.1	104.0±0.3	42.70±5.3	6.06±0.5	2.5±0.05	1.68±0.18
C	72.3±1.2	140.7±0.4	4.3±0.07	102.6±1.1	32.93±2.1	6.37±0.4	2.4±0.03	1.9±0.14

**Figure 1:**
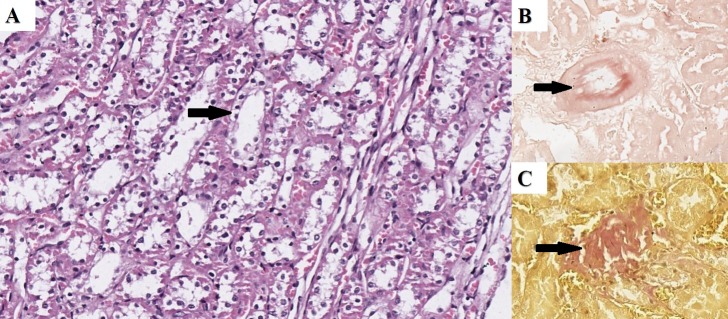
Renal Histopathology in the HFD Group A) Vacuolar degeneration in the epithelial cells covering the tubular system (arrow) – H&E; B) Congo red positive material in the wall of a blood vessel (arrow) – Congo red; C) fibrous tissue in the parenchyma (arrow) – Van-Gieson. Original magnification 200x HFD: high-fructose drinking; H&E: hematoxylin and eosin

Under the H&E stain, the kidney of the HFD rats revealed minimal changes in glomerular composition and cellularity without any diagnostic weight. However, in the tubular section, both the proximal and distal sections revealed visible cellular degeneration with vacuolization in the proximal section and degeneration in the distal section and visible vascular ruptures with perivascular scarring, evident of atherosclerotic changes. These changes were not observed in the control groups (Figures 1A-1C).

**Figure 2:**
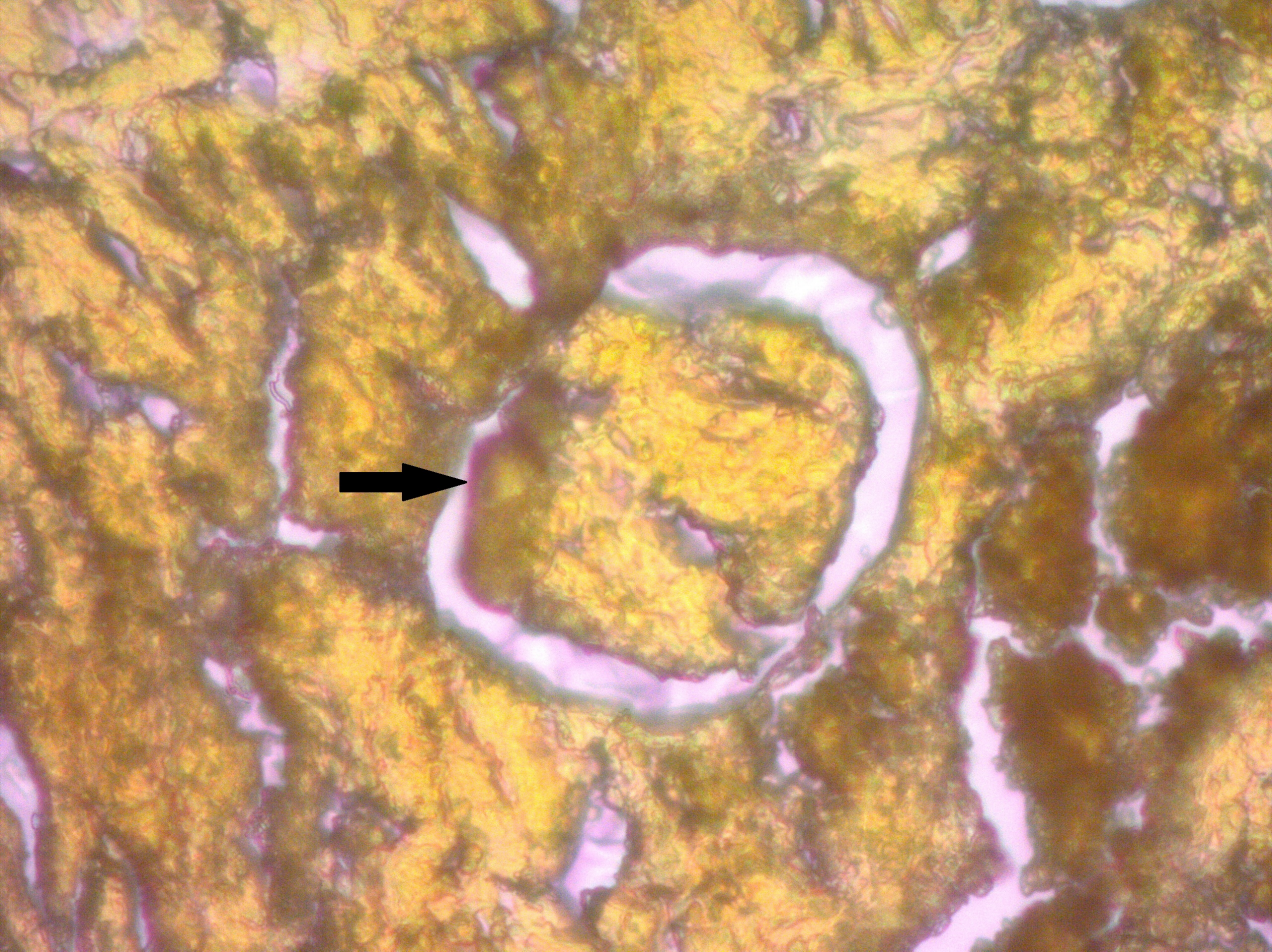
LISA Stain Showing Amyloid Deposits in Glomerulus (arrow) Original magnification 400x. Lugol’s iodine for staining amyloid: LISA

**Figure 3:**
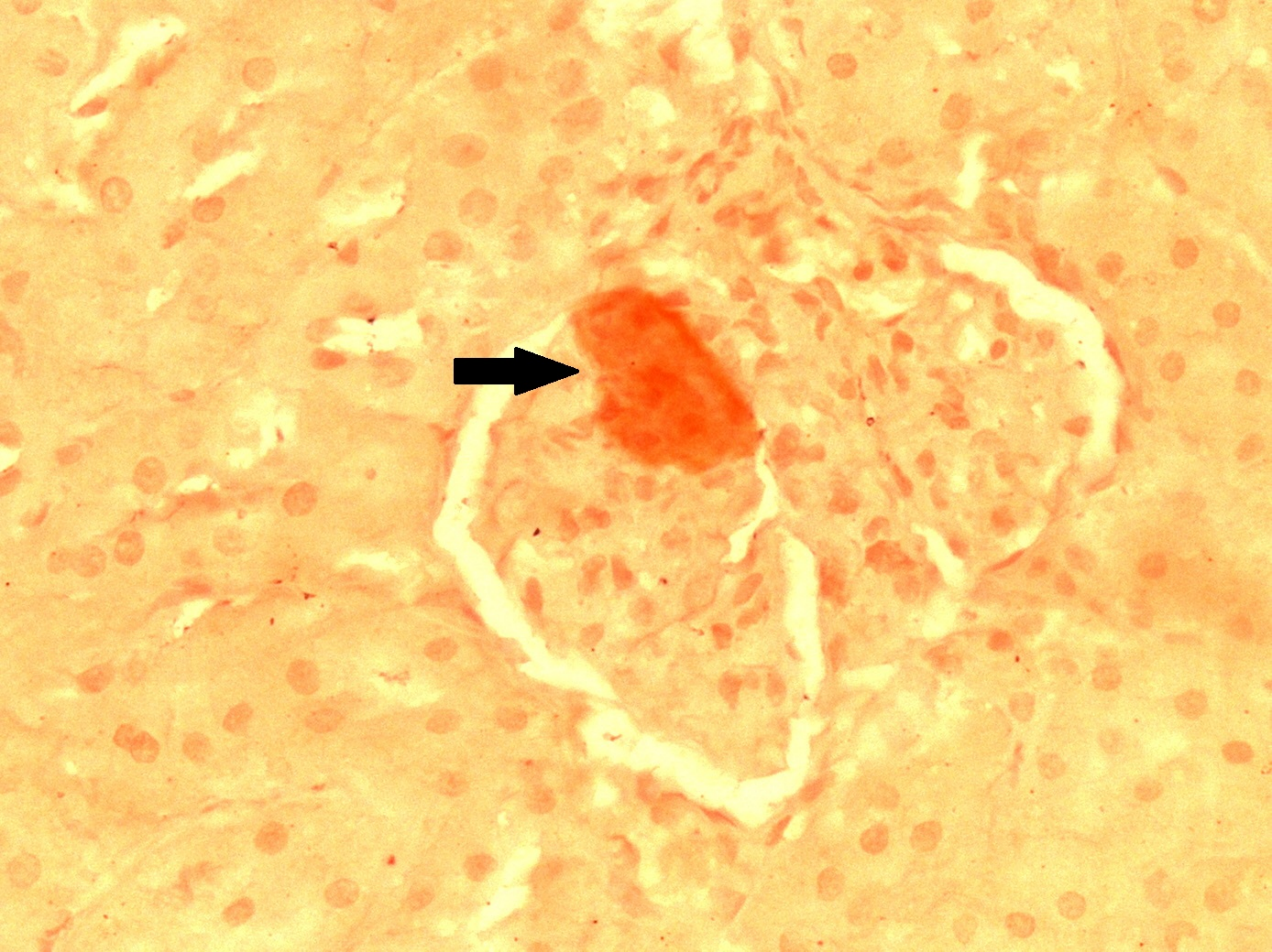
Congo Red Stain Showing Amyloid-positive Deposit in Glomerulus (arrow) Original magnification 400x

On both the Congo red and LISA stains, the kidneys of the HFD rats revealed Kimmelstiel-Wilson-like nodular formations positive for amyloid in the glomeruli and amyloid-positive deposits in the subendothelial layer of the larger blood vessels (Figures 1B, 2-3).

On the Van-Gieson stain, the HFD group revealed parenchymal and perivascular scarring (Figure 1C).

## Discussion

Increased consumption of foods high in fructose correlates with the increased incidence of diabetes, cardiovascular disease, and MS worldwide [11]. Elevated serum uric acid levels are thought to be a potential mechanism linking fructose consumption to this epidemic [12]. Fructose, when metabolized in the liver (unlike other carbohydrates) rapidly increases UA production but also reduces uric acid excretion through the kidneys, which is associated with the induced by it insulin resistance [13]. Nakagawa, et al. found that decreasing the UA level with allopurinol and uricosuric agents affected insulin sensitivity as well as other MS characteristics, such as hypertriglyceridemia, arterial hypertension, and obesity in fructose-fed rodents [14]. Similar studies in patients with arterial hypertension, kidney disease, Type 2 diabetes mellitus, and obesity have shown that hyperuricemia is an independent risk factor for these diseases, and lowering serum UA level significantly improves endothelial function [15].

In our study, the 16 weeks of fructose loading in rats resulted in obesity, hyperglycemia, hypertriglyceridemia, systemic oxidative stress, and an almost twofold increase in UA and CRP levels, which is probably closely related to the morphological changes in the kidneys. Histopathology of the kidneys showed amyloid deposits in the glomeruli, tubulointerstitial injuries, and microvascular lesions with early atherosclerosis prior to developing marked renal dysfunction.

Increased uric acid production is known to lower the level of endothelial nitric oxide (NO), resulting in reduced insulin utilization by skeletal muscles and endothelial dysfunction [16]. On the other hand, by elevating reduced nicotinamide adenine dinucleotide phosphate (NADPH)-oxidase in adipocytes, UA decreases adiponectin secretion and stimulates insulin resistance in obesity. At the same time, in endothelial and smooth muscle cells, the activated NADPH-oxidase increases reactive oxygen species (ROS) production [15, 17-18]. UA induces endothelial dysfunction not only by reducing the vasodilating NO in the endothelium but also by activating the expression of endothelin-1 (ET-1) and cyclooxygenase-2 (COX-2) in the vascular smooth muscle cells, thereby increasing the production of thromboxane and other vasoconstrictor prostaglandins [19]. In addition, UA increases the expression of monocyte chemotactic factor-1 (MCP-1), a chemokine which stimulates macrophage infiltration of atheromatous vessels, cyclic guanosine monophosphate (cGMP) proliferation, and extracellular matrix modification, etc., as well as vascular wall remodeling [20]. Considered together, these processes point to the pathogenic effects of hyperuricemia, which are likely to be of essential importance for the vascular complications found during our experiment.

The metabolic disorders in obesity and insulin resistance in fructose loading cause abnormalities in the lipid metabolism, as evidenced by the elevated glucose and triglyceride levels found in this study, as well as in a number of other investigations [21]. The accumulation of excess lipids in fatty tissue in obesity leads to adipocyte dysfunction, hypoxia, oxidative stress, and chronic inflammation, accompanied by the release of proinflammatory and diabetogenic cytokines, such as tumor necrosis factor alpha (TNF-α), interleukin 6 (IL6), etc. [22]. At the same time, the increased production of ROS elevates the levels of lipid peroxides and their products (such as MDA) throughout the entire body, particularly in the vascular endothelium and liver. Thus, activating stress-sensitive signaling pathways, including the nuclear factor kappa-light-chain-enhancer of activated B cells (NF-kB), P38 mitogen-activated protein kinases (p38 MAPK), including protein kinase C, also increases the production of TNF-α, acute-phase CRP, fibrinogen, and beta cell dysfunction [16]. However, fructose-induced disturbances of lipid metabolism also cause hypertriglyceridemia and thus increase the risk of developing atherosclerosis and hypertension [23].

Increased hepatic production of CRP and fibrinogen has been identified as a key element of MS, associated with the pathogenesis of atherosclerosis [24]. In a number of studies, serum CRP levels have been found to correlate with the degree of low-grade vascular inflammation in people with obesity and MS, which links hyperuricemia and vascular injury [25]. Data from our previous studies demonstrate that the administration of allopurinol reduces oxidative stress in adipose tissue as well as serum UA levels, glucose, and TNF-α in high-fructose feeding, which also corroborates the proinflammatory effect of uric acid [26]. The high serum levels of CRP and UA, and possibly other proinflammatory factors, contribute to the formation and deposition of immune complexes and amyloid in the kidneys, pancreas, and other organs, impairing their functions. Renal amyloidosis causes increased glomerular secretion, stress, and subsequent epithelial degeneration of tubular cells with consequent progressive functional renal impairment, which suggests its association with the development and severity of CKD progression [27-29]. Permanently elevated glucose levels also cause oxidative stress, especially due to the formation of glycosylated products, glucose autooxidation, and sorbitol production, which cause many of the diabetic vascular complications, including nephropathy [30].

## Conclusions

In conclusion, our results show that under conditions of fructose-induced metabolic syndrome and high serum UA and CRP levels, early renal impairment develops (characterized by tubulointerstitial injuries, arteriolopathy, and deposition of amyloid localized in the glomeruli) without any marked manifestation of renal dysfunction. Although the exact mechanisms by which chronic inflammation and oxidative stress in MS damage the kidneys have not been elucidated, we assume that monitoring of serum UA values, along with those of inflammatory and atherogenic markers in MS patients, is of particular importance for assessing the risk of developing CKD.
